# Penikisite, BaMg_2_Al_2_(PO_4_)_3_(OH)_3_, isostructural with bjarebyite

**DOI:** 10.1107/S1600536812051793

**Published:** 2013-01-09

**Authors:** Michael G. Bowman, Robert T. Downs, Hexiong Yang

**Affiliations:** a Department of Geosciences, University of Arizona, 1040 E. 4th Street, Tucson, Arizona 85721-0077, USA

## Abstract

The bjarebyite group of minerals, characterized by the general formula Ba*X*_2_ *Y*_2_(PO_4_)_3_(OH)_3_, with *X* = Mg, Fe^2+^ or Mn^2+^, and *Y* = Al or Fe^3+^, includes five members: bjarebyite BaMn^2+^_2_Al_2_(PO_4_)_3_(OH)_3_, johntomaite BaFe^2+^_2_Fe^3+^_2_(PO_4_)_3_(OH)_3_, kulanite BaFe^2+^_2_Al_2_(PO_4_)_3_(OH)_3_, penikisite BaMg_2_Al_2_(PO_4_)_3_(OH)_3_, and perloffite BaMn^2+^_2_Fe^3+^_2_(PO_4_)_3_(OH)_3_. Thus far, the crystal structures of all minerals in the group, but penikisite, have been determined. The present study reports the first structure determination of penikisite (barium dimagnesium dialuminium triphosphate trihydroxide) using single-crystal X-ray diffraction data of a crystal from the type locality, Mayo Mining District, Yukon Territory, Canada. Penikisite is isotypic with other members of the bjarebyite group with space group *P*2_1_/*m*, rather than triclinic (*P*1 or *P*-1), as previously suggested. Its structure consists of edge-shared [AlO_3_(OH)_3_] octa­hedral dimers linking *via* corners to form chains along [010]. These chains are decorated with PO_4_ tetra­hedra (one of which has site symmetry *m*) and connected along [100] *via* edge-shared [MgO_5_(OH)] octa­hedral dimers and eleven-coordinated Ba^2+^ ions (site symmetry *m*), forming a complex three-dimensional network. O—H⋯O hydrogen bonding provides additional linkage between chains. Microprobe analysis of the crystal used for data collection indicated that Mn substitutes for Mg at the 1.5% (apfu) level.

## Related literature

For penikisite, see: Mandarino *et al.* (1977 ▶). For other mineral members in the bjarebyite group, see: Moore & Araki (1974 ▶); Cooper & Hawthorne (1994 ▶); Kolitsch *et al.* (2000 ▶); Elliott & Willis (2011 ▶). For the definition of polyhedral distortion, see: Robinson *et al.* (1971 ▶).

## Experimental

### 

#### Crystal data

BaMg_2_Al_2_(PO_4_)_3_(OH)_3_
*M* *_r_* = 576.77
Monoclinic, 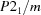

*a* = 8.9577 (4) Å
*b* = 12.0150 (5) Å
*c* = 4.9079 (2) Å
β = 100.505 (2)°
*V* = 519.37 (4) Å^3^
*Z* = 2
Mo *K*α radiation
μ = 4.72 mm^−1^
*T* = 293 K
0.09 × 0.09 × 0.08 mm

#### Data collection

Bruker APEXII CCD diffractometer
Absorption correction: multi-scan (*SADABS*; Sheldrick, 2005 ▶) *T*_min_ = 0.676, *T*_max_ = 0.704
7681 measured reflections
1970 independent reflections
1925 reflections with *I* > 2σ(*I*)
*R*_int_ = 0.020

#### Refinement

*R*[*F* ^2^ > 2σ(*F* ^2^)] = 0.015
*wR*(*F* ^2^) = 0.039
*S* = 1.14
1970 reflections
119 parameters
1 restraint
All H-atom parameters refined
Δρ_max_ = 0.72 e Å^−3^
Δρ_min_ = −0.80 e Å^−3^

### 

Data collection: *APEX2* (Bruker, 2004 ▶); cell refinement: *SAINT* (Bruker, 2004 ▶); data reduction: *SAINT*; program(s) used to solve structure: *SHELXS97* (Sheldrick, 2008 ▶); program(s) used to refine structure: *SHELXL97* (Sheldrick, 2008 ▶); molecular graphics: *XtalDraw* (Downs & Hall-Wallace, 2003 ▶); software used to prepare material for publication: *publCIF* (Westrip, 2010 ▶).

## Supplementary Material

Crystal structure: contains datablock(s) I, New_Global_Publ_Block. DOI: 10.1107/S1600536812051793/hb7009sup1.cif

Structure factors: contains datablock(s) I. DOI: 10.1107/S1600536812051793/hb7009Isup2.hkl

Additional supplementary materials:  crystallographic information; 3D view; checkCIF report

## Figures and Tables

**Table 1:** Selected bond lengths (Å)

Mg—O7^i^	2.0591 (11)
Mg—O1^ii^	2.0864 (10)
Mg—O2^iii^	2.1227 (10)
Mg—O*H*9^iv^	2.1729 (11)
Mg—O5^v^	2.2090 (12)
Al—O3^vi^	1.8523 (11)
Al—O6	1.9080 (11)
Al—O5^vii^	1.9287 (10)
Al—O*H*9	1.9397 (11)
Al—O*H*9^viii^	1.9440 (11)
Al—O*H*8	1.9477 (7)
P1—O2	1.5232 (14)
P1—O1	1.5278 (15)
P1—O3	1.5321 (10)
P1—O3^ix^	1.5321 (10)
P2—O4	1.5083 (11)
P2—O7	1.5272 (11)
P2—O6	1.5443 (11)
P2—O5	1.5680 (10)

Symmetry codes: (i) 
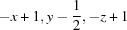
; (ii) 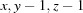; (iii) 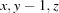; (iv) 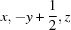; (v) 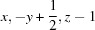; (vi) 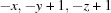; (vii) 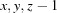; (viii) 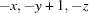; (ix) 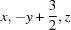.

**Table 2:** Hydrogen-bond geometry (Å, °)

*D*—H⋯*A*	*D*—H	H⋯*A*	*D*⋯*A*	*D*—H⋯*A*
O*H*8—H1⋯O6^vii^	0.79 (4)	2.66 (3)	3.3180 (16)	142 (1)
O*H*9—H2⋯O3	0.78 (3)	1.89 (3)	2.6512 (13)	166 (3)

Symmetry code: (vii) 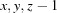.

**Fig. 1.**
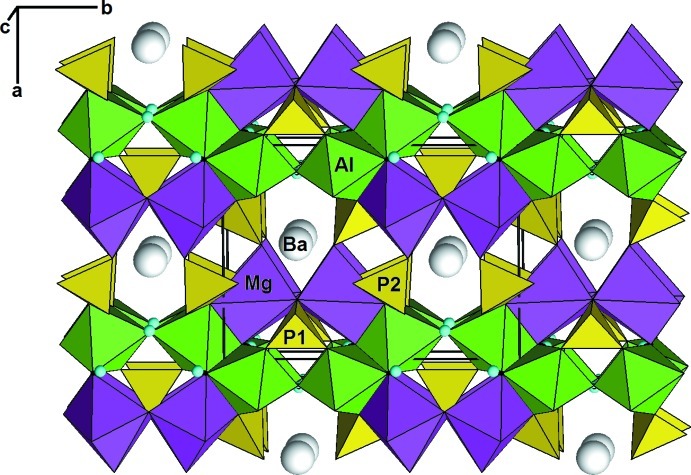
Crystal structure of penikisite in polyhedral representation. Large and small spheres represent Ba and H atoms, respectively.

**Fig. 2.**
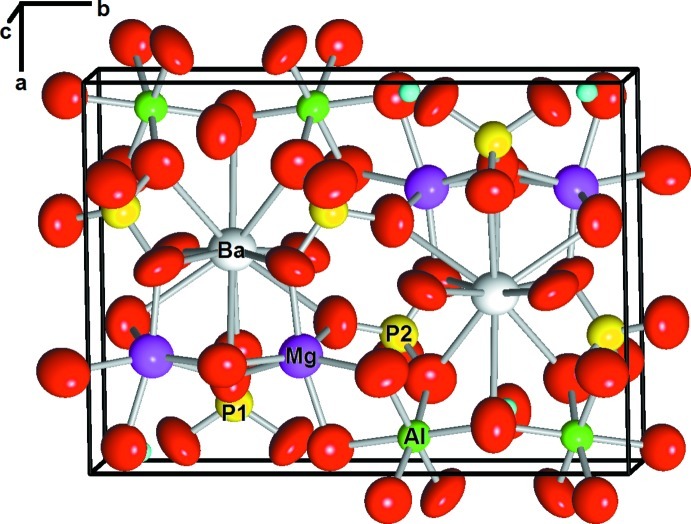
The crystal structure of penikisite, showing atoms, except for H, with displacement ellipsoids at the 99% probability level. Gray, pink, green, yellow, and red ellipsoids represent Ba, Mg, Al, P, and O atoms, respectively. H atoms are given as turquoise spheres with an arbitrary radius.

## supplementary crystallographic information

### Comment

The bjarebyite group of minerals is characterized by the general chemical formula Ba*X*_2_*Y*_2_(PO_4_)_3_(OH)_3_, where *X*=Mn^2+^, Fe^2+^ or Mg and *Y*=Al or Fe^3+^, and includes five members: bjarebyite BaMn^2+^_2_Al_2_(PO_4_)_3_(OH)_3_, johntomaite BaFe^2+^_2_Fe^3+^_2_(PO_4_)_3_(OH)_3_, kulanite BaFe^2+^_2_Al_2_(PO_4_)_3_(OH)_3_, penikisite BaMg_2_Al_2_(PO_4_)_3_(OH)_3_, and perloffite BaMn^2+^_2_Fe^3+^_2_(PO_4_)_3_(OH)_3_. Except for penikisite, the crystal structures of all other minerals in the group have been determined (Moore and Araki, 1974; Cooper and Hawthorne, 1994; Kolitsch *et al.*, 2000; Elliot & Willis, 2011), which all possess space group *P*2_1_/*m*. Penikisite was first described by Mandarino *et al.* (1977) as triclinic with space group *P*1 or *P*1 (albeit strongly pseudomonoclinic), based on the observation of asymmetric optical dispersion. Since then, no detailed crystallographic study on penikisite has been reported. In our efforts to understand the hydrogen bonding environments in minerals, we conducted a structure determination of penikisite from the type locality by means of single-crystal X-ray diffraction.

Penikisite is isotypic with other members of the bjarebyite group, with space group *P*2_1_/*m*. Its structure consists of edge-shared [AlO_3_(OH)_3_] octahedral dimers connected *via* corners to form chains along [010]. These chains are decorated with PO_4_ tetrahedra and linked along [100] *via* edge-shared MgO_5_(OH) octahedral dimers and eleven-coordinated Ba atoms to form a complex three-dimensional network (Figs. 1 and 2). The hydrogen bonding provides additional linkage between chains.

Similar to other minerals in the bjarebyite group, the *Y*O_5_(OH) octahedra in penikisite are noticeably distorted, as measured by the octahedral angle variance (OAV) and quadratic elongation (OQE) (Robinson *et al.*, 1971), which are 188 and 1.057, respectively. In contrast, the OAV and OQE values are 32 and 1.010 for the *X*O_3_(OH)_3_ octahedra in penikisite. From penikisite to the Fe-analogue kulanite (Cooper and Hawthorne, 1994), and to the Mn-analogue bjarebyite (Moore and Araki, 1974), the average *X*-O distance increases from 2.117 to 2.146, and to 2.162 Å, respectively, in accordance with the increase in the ionic radius in this site.

There are two hydrogen bonds in penikisite, one between OH8 and O6 [3.318 (2) Å] and the other between OH9 and O3 [2.651 (1) Å], agreeing well with the results obtained by Elliott & Willis (2011) from perloffite. However, Cooper and Hawthorne (1994) proposed a disorder model for H1 in kulanite. The H atoms were not located in the structure of bjarebyite (Moore and Araki, 1974) or johntomaite (Kolitsch *et al.*, 2000).

### Experimental

The penikisite crystal used in this study is from the type locality, 16 miles north of the Hess River, Mayo Mining District, Yukon Territory, Canada and is in the collection of the RRUFF project (http://rruff.info/R060160), donated by Mark Mauthner. Its chemistry was determined with a CAMECA SX50 electron microprobe (8 analysis points), yielding the empirical chemical formula, calculated on the basis of 13.5 O atoms, Ba_1.00_(Mg_1.97_Mn_0.03_)_Σ__=2_Al_2.00_(P_1.00_O_4_)_3_(OH)_3_ (OH was estimated by charge balance and difference).

### Refinement

The H atoms were located from difference Fourier syntheses and their positions refined freely with a fixed isotropic displacement (*U_iso_* = 0.03). The highest residual peak in the difference Fourier maps was located at (0.4023, 0.2932, 0.2033), 0.71 Å from Ba, and the deepest hole at (0.5192, 1/4, 0.3234), 0.63 Å from Ba.

### Crystal data

**Table:**

Al_4_H_6_Mg_3.94_Mn_0.06_O_30_P_6_·2(Ba)	*F*(000) = 549
*M**_r_* = 576.77	nearly cube
Monoclinic, *P*12_1_/*m*1	*D*_x_ = 3.688 Mg m^−^^3^
Hall symbol: -P 2yb	Mo *K*α radiation, λ = 0.71073 Å
*a* = 8.9577 (4) Å	Cell parameters from 6030 reflections
*b* = 12.0150 (5) Å	θ = 2.9–32.6°
*c* = 4.9079 (2) Å	µ = 4.72 mm^−^^1^
β = 100.505 (2)°	*T* = 293 K
*V* = 519.37 (4) Å^3^	Cube, green
*Z* = 2	0.09 × 0.09 × 0.08 mm

### Data collection

**Table:**

Bruker APEXII CCD diffractometer	1970 independent reflections
Radiation source: fine-focus sealed tube	1925 reflections with *I* > 2σ(*I*)
Graphite monochromator	*R*_int_ = 0.020
φ and ω scan	θ_max_ = 32.6°, θ_min_ = 2.9°
Absorption correction: multi-scan (*SADABS*; Sheldrick 2005)	*h* = −13→12
*T*_min_ = 0.676, *T*_max_ = 0.704	*k* = −15→18
7681 measured reflections	*l* = −7→7

### Refinement

**Table:**

Refinement on *F*^2^	Secondary atom site location: difference Fourier map
Least-squares matrix: full	Hydrogen site location: difference Fourier map
*R*[*F*^2^ > 2σ(*F*^2^)] = 0.015	All H-atom parameters refined
*wR*(*F*^2^) = 0.039	*w* = 1/[σ^2^(*F*_o_^2^) + (0.020*P*)^2^ + 0.2753*P*] where *P* = (*F*_o_^2^ + 2*F*_c_^2^)/3
*S* = 1.14	(Δ/σ)_max_ = 0.001
1970 reflections	Δρ_max_ = 0.72 e Å^−^^3^
119 parameters	Δρ_min_ = −0.80 e Å^−^^3^
1 restraint	Extinction correction: *SHELXL*, Fc^*^=kFc[1+0.001xFc^2^λ^3^/sin(2θ)]^-1/4^
Primary atom site location: structure-invariant direct methods	Extinction coefficient: 0.0021 (5)

### Special details

**Table:**

Geometry. All e.s.d.'s (except the e.s.d. in the dihedral angle between two l.s. planes) are estimated using the full covariance matrix. The cell e.s.d.'s are taken into account individually in the estimation of e.s.d.'s in distances, angles and torsion angles; correlations between e.s.d.'s in cell parameters are only used when they are defined by crystal symmetry. An approximate (isotropic) treatment of cell e.s.d.'s is used for estimating e.s.d.'s involving l.s. planes.
Refinement. Refinement of *F*^2^ against ALL reflections. The weighted *R*-factor *wR* and goodness of fit *S* are based on *F*^2^, conventional *R*-factors *R* are based on *F*, with *F* set to zero for negative *F*^2^. The threshold expression of *F*^2^ > σ(*F*^2^) is used only for calculating *R*-factors(gt) *etc*. and is not relevant to the choice of reflections for refinement. *R*-factors based on *F*^2^ are statistically about twice as large as those based on *F*, and *R*- factors based on ALL data will be even larger.

### Fractional atomic coordinates and isotropic or equivalent isotropic displacement parameters (Å^2^)

**Table:**

	*x*	*y*	*z*	*U*_iso_*/*U*_eq_	Occ. (<1)
Ba	0.547869 (12)	0.7500	0.74171 (2)	0.00734 (5)	
Mg	0.29439 (5)	−0.11139 (4)	0.20677 (10)	0.00705 (9)	0.9850 (1)
Mn	0.29439 (5)	−0.11139 (4)	0.20677 (10)	0.00705 (9)	0.0150 (1)
Al	0.09176 (4)	0.40084 (3)	0.12947 (8)	0.00401 (8)	
P1	0.15736 (6)	0.7500	0.68481 (10)	0.00467 (9)	
P2	0.33413 (4)	0.44282 (3)	0.70566 (7)	0.00495 (7)	
O1	0.27909 (16)	0.7500	0.9471 (3)	0.0072 (2)	
O2	0.23251 (16)	0.7500	0.4303 (3)	0.0068 (2)	
O3	0.05983 (11)	0.64525 (9)	0.6850 (2)	0.00791 (18)	
O4	0.36649 (12)	0.55738 (9)	0.6050 (2)	0.00846 (18)	
O5	0.25965 (11)	0.45434 (9)	0.9697 (2)	0.00811 (18)	
O6	0.22678 (12)	0.38012 (9)	0.4741 (2)	0.00917 (18)	
O7	0.47653 (12)	0.37189 (9)	0.7901 (2)	0.00821 (18)	
OH8	0.12478 (17)	0.2500	0.0077 (3)	0.0083 (3)	
OH9	0.06100 (12)	0.55814 (9)	0.1891 (2)	0.00679 (17)	
H1	0.137 (4)	0.2500	−0.147 (8)	0.030*	
H2	0.046 (3)	0.585 (2)	0.325 (5)	0.030*	

### Atomic displacement parameters (Å^2^)

**Table:**

	*U*^11^	*U*^22^	*U*^33^	*U*^12^	*U*^13^	*U*^23^
Ba	0.00667 (6)	0.00826 (7)	0.00724 (6)	0.000	0.00171 (4)	0.000
Mg	0.0069 (2)	0.0070 (2)	0.00715 (19)	0.00042 (16)	0.00119 (16)	−0.00064 (16)
Mn	0.0069 (2)	0.0070 (2)	0.00715 (19)	0.00042 (16)	0.00119 (16)	−0.00064 (16)
Al	0.00373 (17)	0.00395 (17)	0.00440 (16)	−0.00027 (13)	0.00091 (13)	0.00002 (13)
P1	0.00489 (19)	0.0050 (2)	0.00428 (18)	0.000	0.00138 (15)	0.000
P2	0.00499 (14)	0.00536 (15)	0.00459 (14)	0.00024 (11)	0.00113 (11)	0.00027 (10)
O1	0.0072 (6)	0.0085 (6)	0.0055 (6)	0.000	0.0001 (5)	0.000
O2	0.0088 (6)	0.0061 (6)	0.0062 (6)	0.000	0.0036 (5)	0.000
O3	0.0082 (4)	0.0069 (4)	0.0095 (4)	−0.0029 (3)	0.0040 (3)	−0.0015 (3)
O4	0.0101 (4)	0.0070 (4)	0.0083 (4)	−0.0001 (3)	0.0018 (3)	0.0023 (3)
O5	0.0084 (4)	0.0102 (5)	0.0067 (4)	−0.0008 (4)	0.0037 (3)	−0.0006 (3)
O6	0.0093 (4)	0.0100 (5)	0.0073 (4)	−0.0002 (4)	−0.0008 (3)	−0.0015 (3)
O7	0.0058 (4)	0.0087 (5)	0.0102 (4)	0.0021 (3)	0.0018 (3)	0.0020 (3)
OH8	0.0110 (6)	0.0071 (6)	0.0071 (6)	0.000	0.0027 (5)	0.000
OH9	0.0078 (4)	0.0076 (4)	0.0049 (4)	−0.0001 (3)	0.0012 (3)	−0.0016 (3)

### Geometric parameters (Å, º)

**Table:**

Ba—O7^i^	2.7669 (10)	Mg—O5^x^	2.2090 (12)
Ba—O7^ii^	2.7669 (10)	Al—O3^xi^	1.8523 (11)
Ba—O1	2.7744 (14)	Al—O6	1.9080 (11)
Ba—O4	2.8370 (11)	Al—O5^xii^	1.9287 (10)
Ba—O4^iii^	2.8370 (11)	Al—OH9	1.9397 (11)
Ba—O6^iv^	2.9019 (11)	Al—OH9^xiii^	1.9440 (11)
Ba—O6^v^	2.9019 (10)	Al—OH8	1.9477 (7)
Ba—O2	2.9566 (15)	P1—O2	1.5232 (14)
Ba—OH8^v^	2.9658 (15)	P1—O1	1.5278 (15)
Ba—O7^v^	2.9661 (10)	P1—O3	1.5321 (10)
Ba—O7^iv^	2.9661 (10)	P1—O3^iii^	1.5321 (10)
Mg—O4^vi^	2.0490 (11)	P2—O4	1.5083 (11)
Mg—O7^vii^	2.0591 (11)	P2—O7	1.5272 (11)
Mg—O1^viii^	2.0864 (10)	P2—O6	1.5443 (11)
Mg—O2^ix^	2.1227 (10)	P2—O5	1.5680 (10)
Mg—OH9^vi^	2.1729 (11)		
			
O4^vi^—Mg—O7^vii^	83.31 (4)	O3^xi^—Al—OH9^xiii^	89.97 (5)
O4^vi^—Mg—O1^viii^	144.07 (5)	O6—Al—OH9^xiii^	170.24 (5)
O7^vii^—Mg—O1^viii^	83.17 (5)	O5^xii^—Al—OH9^xiii^	94.31 (5)
O4^vi^—Mg—O2^ix^	79.75 (5)	OH9—Al—OH9^xiii^	77.04 (5)
O7^vii^—Mg—O2^ix^	105.87 (5)	O3^xi^—Al—OH8	92.21 (5)
O1^viii^—Mg—O2^ix^	72.26 (5)	O6—Al—OH8	92.49 (6)
O4^vi^—Mg—OH9^vi^	94.50 (4)	O5^xii^—Al—OH8	90.69 (5)
O7^vii^—Mg—OH9^vi^	168.32 (5)	OH9—Al—OH8	170.59 (5)
O1^viii^—Mg—OH9^vi^	104.78 (5)	OH9^xiii^—Al—OH8	96.50 (6)
O2^ix^—Mg—OH9^vi^	84.93 (5)	O2—P1—O1	109.67 (8)
O4^vi^—Mg—O5^x^	102.80 (5)	O2—P1—O3	109.74 (5)
O7^vii^—Mg—O5^x^	97.57 (4)	O1—P1—O3	108.60 (5)
O1^viii^—Mg—O5^x^	111.89 (4)	O2—P1—O3^iii^	109.74 (5)
O2^ix^—Mg—O5^x^	156.55 (5)	O1—P1—O3^iii^	108.60 (5)
OH9^vi^—Mg—O5^x^	71.65 (4)	O3—P1—O3^iii^	110.46 (8)
O3^xi^—Al—O6	85.88 (5)	O4—P2—O7	113.41 (6)
O3^xi^—Al—O5^xii^	174.52 (5)	O4—P2—O6	109.59 (6)
O6—Al—O5^xii^	89.36 (5)	O7—P2—O6	107.74 (6)
O3^xi^—Al—OH9	94.60 (5)	O4—P2—O5	109.04 (6)
O6—Al—OH9	94.47 (5)	O7—P2—O5	106.57 (6)
O5^xii^—Al—OH9	83.07 (5)	O6—P2—O5	110.45 (6)

Symmetry codes: (i) −*x*+1, *y*+1/2, −*z*+2; (ii) −*x*+1, −*y*+1, −*z*+2; (iii) *x*, −*y*+3/2, *z*; (iv) −*x*+1, *y*+1/2, −*z*+1; (v) −*x*+1, −*y*+1, −*z*+1; (vi) *x*, −*y*+1/2, *z*; (vii) −*x*+1, *y*−1/2, −*z*+1; (viii) *x*, *y*−1, *z*−1; (ix) *x*, *y*−1, *z*; (x) *x*, −*y*+1/2, *z*−1; (xi) −*x*, −*y*+1, −*z*+1; (xii) *x*, *y*, *z*−1; (xiii) −*x*, −*y*+1, −*z*.

### Hydrogen-bond geometry (Å, º)

**Table:**

*D*—H···*A*	*D*—H	H···*A*	*D*···*A*	*D*—H···*A*
O*H*8—H1···O6^xii^	0.79 (4)	2.66 (3)	3.3180 (16)	142 (1)
O*H*9—H2···O3	0.78 (3)	1.89 (3)	2.6512 (13)	166 (3)

Symmetry code: (xii) *x*, *y*, *z*−1.
